# The Story of the Insane from Year to Year

**Published:** 1896-10-31

**Authors:** 


### THE STORY OF THE INSANE FROM YEAR TO YEAR.

Ninth Series.

### THE SUFFOLK COUNTY ASYLUM.

On January 1st, 1895, this asylum contained 551 patients, and on December 31st the number had risen to 589. There were 204 admissions (17 being re-admissions), 71 recoveries, 9 discharged relieved, and 68 deaths. Of the deaths 23 were caused by cerebral and spinal diseases, 15 by consumption, and 3 by pneumonia. The percentage of recoveries calculated on the admissions is 43·23, and that of the deaths is 11·54 on the average number resident. We did not notice any table showing the causes of insanity in the year's admissions. The asylum has passed through an eventful year, and Dr. Eager must have had a very anxious time of it. In addition to dysentery, from which the asylum seems never to be free, there was an outbreak of a curious disease concerning which there was some doubt as to whether it was lead poisoning or Beri Beri; and this epidemic was followed by an outbreak of scarlet fever, from which 33 persons suffered. There were 53 cases of dysentery, of whom 12 died. Dr. Eager remarks that this dysentery is of a most uncommon kind. "Many cases assume all the characteristics of typhoid fever during life; but morbid anatomy shows that we must attribute them to the same cause and treat them as dysentery." There is no doubt, as we have already pointed out in these notices, that there is a disease sometimes seen in asylums which is clinically identical with typhoid, but which is nevertheless not the typhoid seen in our large towns. The asylum is practically full, and it will soon be necessary to decide what to do to relieve or prevent the overcrowding sure to make its appearance. One idea is to build additions for 220 patients, and the other is to build a new asylum for West Suffolk. Were the present building in a good state and adapted for the treatment of the insane, we should say the first course would be the better one; but it is well known not to be up-to-date structurally, therefore there can hardly be a doubt that West Suffolk should strike out a line for itself. The committee say that the general supervision and management of the asylum by the Medical Superintendent has been very satisfactory. The average weekly cost is 10s. per head.

### GLAMORGAN ASYLUM AT BRIDGEND.

There were 1,162 patients on January 1st, 1895, and by December 31st the number had risen to 1,316. The first admissions were 371, the readmissions 35, the recoveries 96, the relieved 48, and the deaths 105. The recoveries were 28·6 per cent. on the admissions, and the deaths were 6·7 per cent. on the average number resident. Of the deaths, 52 were from cerebral and spinal diseases (including 34 from general paralysis), 16 from consumption, and 3 from diarrhœa. Drink is the assigned cause of the insanity in 92 cases, hereditary influence in 119, and moral causes in 68. In other words, not less than 50 per cent. of the admissions were due to preventable causes. We notice that there are in this asylum three paid chaplains to look after the spiritual welfare of 1,300 inmates, most of whom are hopelessly demented. Surely it would be more reasonable if these chaplains were paid to preach to the people of Glamorganshire on the crime of drunkenness and the awful sin of marriage between people with hereditary insane taint. Probably the chaplains would say that this crusade would need a Peter the Hermit to stir them up; but, at least, it might be tried. It is only by opening the eyes of the people that the onward march of this frightful scourge of insanity can be stayed. The wholesale performing of post-mortem examinations, and the preparation of beautiful microscopic slides will do but little in the matter. Dr. Pringle thinks the type of insanity is changing, melancholia becoming less common and mania taking its place, with the result that there is an increase of noise and excitement. As Dr. Pringle truly says, this is very trying and irksome to those in charge; but then so is the watching of suicidal melancholics. We have often stated that if any class of people really deserve a good pension it is those who have the charge of the insane. The committee acknowledge the great devotion displayed by Dr. Pringle. The average weekly cost per head is 8s. 2d.

### NOTTINGHAM BOROUGH ASYLUM.

Here the numbers fell from 597 to 576, but this decrease was owing to the discharge, or rather transference, of chronic cases to the asylum for their own county. There were 155 admissions, including 20 readmissions, 60 recoveries, and 59 deaths. The recoveries were 41·1 per cent. of the admissions, and the deaths were 10 per cent. on the average resident. Drink was the assigned cause in 27 of the year's admissions, and hereditary influence in only 15. It is often difficult to get the friends of patients to admit that there is "insanity in the family," and the number here given (15 out of 155) is manifestly much too low. Of the 59 deaths 11 were due to cerebral and spinal diseases, 3 to consumption, and 3 to bronchitis. Four patients escaped during the year, and Mr. Powell says, "It is well nigh impossible in a modern asylum to cope with a determined runaway without adopting such measures of restraint as would militate very considerably against the welfare of the rest of the patients." This principle is acted upon in nearly all our county asylums, and it would seem fairly certain that the greater the liberty given to the patients the less is the desire to escape. The asylum was opened sixteen years ago, and had extensive additions a few years since. It is now almost full again, and further building will be imperative. Mr. Powell shows that in 1886 the ratio of admissions to the population of the borough was only 4·26 per 10,000, whereas in 1895 it was 6·77; but this must not be taken to prove that insanity has increased to anything like that extent in Nottingham. The weekly charge for maintenance is 10s.

